# The diagnostic performance of neck ultrasound in follow-up of advanced stage differentiated thyroid cancer

**DOI:** 10.1186/s13044-024-00213-8

**Published:** 2024-10-14

**Authors:** Vicki Munro, Syed Mustafa, Ferhan S. Siddiqi, Murali Rajaraman, Andreu F. Costa, Syed Ali Imran

**Affiliations:** 1 Division of Endocrinology, Department of Medicine, Dalhousie University, 7N Victoria Building, 1276 South Park St Halifax, Halifax, NS B3H 2Y9 Canada; 2 Faculty of Medicine, Dalhousie University, Halifax, NS Canada; 3 Department of Diagnostic Radiology, Dalhousie University, Halifax, NS Canada; 4 Department of Radiation Oncology, Dalhousie University, Halifax, NS Canada

**Keywords:** Thyroid cancer, Ultrasound, Thyroglobulin, Prognosis, Recurrence

## Abstract

**Background:**

Differentiated thyroid cancer (DTC) requires long-term follow-up due to the risk of delayed recurrence. Follow-up surveillance involves serial neck ultrasound (US) and thyroglobulin (Tg); however, the optimal frequency and diagnostic performance of neck US outside of specialized thyroid cancer centres in higher risk patients is not well defined. We sought to evaluate the diagnostic performance of US and serial Tg in advanced stage DTC.

**Methods:**

We retrospectively reviewed our thyroid cancer database for patients with stage III and IV DTC from 2006 to 2018, total thyroidectomy, and at least 2 years follow-up to assess recurrence rates. Those with hemi-thyroidectomy or anti-Tg antibodies were excluded. Diagnostic performance of US and Tg were assessed using a composite reference standard of follow-up imaging and pathology. All relevant US were reviewed by a blinded expert radiologist for uniformity.

**Results:**

Of 136 included patients (91 females, mean age 58.9), 26 (19%) had recurrence of DTC over median follow-up of 6.6 years (IQR 5.3–9.3). The sensitivity and specificity of US in diagnosing cervical recurrence were 73.3% (95% CI 0.51–0.96) and 68.3% (95% CI 0.60–0.77) based on historical reports, respectively, and 80% (95% CI 0.60-1.00) and 87.8% (95% CI 0.82–0.93) based on blinded expert review, respectively. Tg had a sensitivity of 95.5% (95% CI 0.89-1.0) and specificity of 96.2% (95% CI 0.92–0.99) in detecting cervical recurrence or distant metastases. False positive US findings on historical US and subsequent review occurred in 38 (28%) and 15 (11%) patients, respectively, while 5 (3.6%) had false positive Tg results.

**Conclusion:**

Serial Tg has better sensitivity and specificity than US for detecting recurrence of advanced stage DTC. Furthermore, re-interpretation of abnormal findings using structured US reporting with a subspecialized reader may improve diagnostic performance of US and improve its utility in clinical care.

## Background

The prevalence of differentiated thyroid cancer (DTC) has rapidly risen globally during the past few decades and is most commonly diagnosed in females aged 15–49 [1]. The rising prevalence of DTC is partly attributed to the increased utility of sensitive imaging techniques such as ultrasound (US) and computerized tomography (CT) which are detecting smaller, early-stage cancers [2]. Although the overall prognosis of DTC is quite favorable with a 10-year survival of 97% [1], patients can experience recurrence even decades later. This rising prevalence combined with excellent survival and significant risk of recurrence has led to a growing population of patients who require lifelong surveillance.

Historically, the cornerstones of long-term surveillance have been thyroglobulin (Tg) monitoring and serial neck US imaging; however, the optimal frequency of follow-up is not well defined. Current guidelines suggest that neck US should be done within 6–12 months of initial treatment, but subsequent monitoring is unclear; the American Thyroid Association suggests “periodic” monitoring based on risk of recurrence [3] whereas the European Thyroid Association (ETA) suggests annual monitoring for the first 5 years in high-risk patients [4]. Additionally, US has high rates of false positive findings (34–67%) causing additional investigations and anxiety for patients [5, 6]. US is user-dependent and associated with considerable inter-user variability even amongst dedicated thyroid imaging experts, with one study demonstrating only 33% concordance in classifying nodules according to EU-TIRADS [7].

There is evidence in low and intermediate risk DTC that routine US is likely unnecessary in the context of undetectable thyroglobulin levels [8]. To date, no study has assessed the utility of US in patients with more advanced DTC. To address this knowledge gap, we assessed the diagnostic performance of neck US and serial Tg in the surveillance of advanced stage DTC, using the Standards for Reporting Diagnostic Accuracy (STARD) [9].

## Materials and methods

The Halifax Interdisciplinary Thyroid Oncology Clinic (ITOC) is the sole tertiary care centre for patients with DTC in the province of Nova Scotia, Canada. DTC patients sign informed consent to have their data prospectively entered into the ITOC registry (established in 2006) and all patients are followed by the same multidisciplinary team of radiation oncologists and endocrinologists post-operatively.

For this study, we conducted a retrospective analysis of patients seen at the ITOC from January 1, 2006 – December 31, 2018, using the following inclusion criteria: (a) Stage III or IV DTC (papillary, follicular, or Hurthle cell thyroid cancer) as per the AJCC/UICC staging system in place at time of diagnosis, (b) prior total thyroidectomy, and (c) at least 2 years of follow up. Patients who had elevated anti-Tg antibodies at initial visit, did not have any US imaging during follow-up, or underwent partial thyroidectomy were excluded. The study was approved by our institutional research ethics board.

The following data were gathered from each clinical visit: US and other subsequent imaging, serum Tg and anti-Tg antibody, and pathologic results from biopsies (if conducted). In addition, we gathered data on age at presentation, sex, initial tumour pathology and subtype variant.

## Index test - ultrasound

Given the structure of healthcare within our province, it is not feasible for patients to travel to the tertiary care academic centre for each US examination. As such, US are performed at the nearest available hospital (which includes 2 academic and 11 community sites) and predominantly reported by radiologists without subspecialty training in US. Because of this heterogeneity, we evaluated US performance in two ways: using the historical reports of US examinations, using the US examination closest to the date of confirmed recurrence when applicable or when false positive results resulted in further investigations; and retrospective reinterpretation of the representative US by a subspecialty radiologist (AFC) with 9 years of post-fellowship experience. The subspecialty radiologist was blinded to the original US report, prior US examinations, patient history and outcomes. Images available to the original radiologist were used; prior to 2016 these were static images, while after 2016 included cine clips. These retrospective reviews were done according to the ETA standardized reporting system [4], reporting on size, shape, borders, echogenicity, internal consistency, and vascularization of thyroid bed lesions and size, shape, echogenicity, microcalcifications, and Doppler US features of lymph nodes. Detected thyroid bed and LN lesions were classified as “negative”, “indeterminate”, or “positive” in each. Indeterminate and positive results were considered abnormal findings for analysis in the 2 × 2 contingency table.

### Index test – serum thyroglobulin

Serum Tg was measured with a chemiluminescent immunometric assay (Simens Immulite 2000 XPI) until 2020 when it was replaced with high sensitivity Tg (HS-Tg) assay (Roche e-411). Prior to the implementation of HS-Tg, recombinant TSH (rTSH) stimulated Tg was arranged at the first visit (6–9 months after completion of therapy) for those who received I-131 ablation and if in an indeterminate range (*≤* 10 ng/mL), then additional rTSH Tg testing was arranged in the first 18–24 months in accordance with contemporaneous guidelines [3]. If stimulated Tg was overtly elevated (> 10 ng/mL) then subsequent Tg testing was done without TSH stimulation. All subsequent Tg testing was performed unstimulated.

### Reference standard

A composite reference standard based on imaging and pathology was used. Cervical recurrence was defined as imaging evidence of disease with either positive tissue diagnosis on fine needle aspirate biopsy (FNAB) or surgical pathology, and distant metastases defined as rising Tg with structural abnormalities detected on anatomic (CT/MRI) and/or functional imaging studies (I-131 whole body scan [WBS] or PET scan). FNAB was pursued at the discretion of the treating team, usually in the context of multidisciplinary case round discussion. False positive US result was defined as findings reported as suspicious by the radiologist which were biopsy proven to be negative for recurrence or spontaneously resolved on subsequent imaging without intervention. While in clinical practice those with elevated Tg would be considered “biochemically incomplete”, for the purpose of this study, false positive Tg was defined as Tg rising continuously over three or more visits without evidence of disease on anatomical/functional imaging such as CT scan, I-131 WBS or PET scan.

### Statistical analysis

Categorical variables were expressed as numbers and percentages and continuous variables as mean and standard deviation for normally distributed data, median and interquartile range, and counts and percentages when appropriate. Sensitivity and specificity of ultrasound and Tg in detecting recurrence with 95% confidence intervals (CIs) were calculated. Those with distant metastases only were categorized as “no recurrence” for calculating ultrasound sensitivity and specificity in detecting cervical recurrence but included as “true recurrence” for calculating thyroglobulin sensitivity and specificity. IBM SPSS statistics software version 28.0 was used for analysis.

## Results

**Fig. 1:**
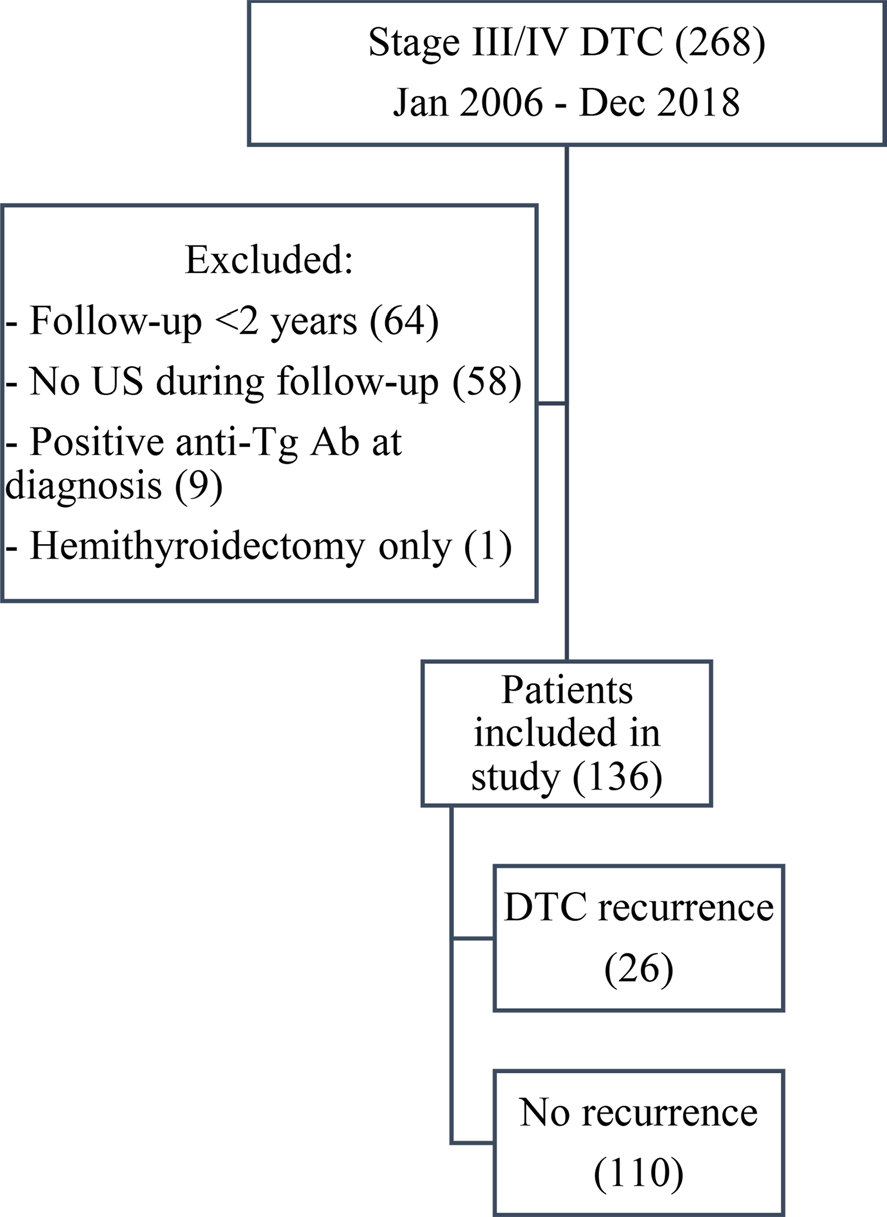
Flow of participants

A total of 268 patients with stage III and IV DTC were screened; 136 patients fulfilled the inclusion criteria (Fig. 1). The patient demographics, tumour details, and treatment information are summarized in Table 1. Median duration of follow-up was 6.6 (IQR 5.3–9.3) years. Recurrence was detected in 26 (19%) patients during the follow-up period. Median time to recurrence was 2.4 (IQR 1.4–5.2) years. Sites of recurrence included 14 cervical region (5 thyroid bed, 9 lymph node), 2 concomitant cervical and distant metastases (1 thyroid bed and humerus, and 1 lymph node and lung), and 10 distant metastases alone (6 lung, 1 mediastinal mass, 1 skeletal [lumbar vertebrae], 1 adrenal gland, and 1 both skeletal and lung).

**Table 1:** Baseline characteristics of DTC patients

Characteristics	
Age (years) mean (SD)	58.9 (9.8)
Females n (%)	91 (67)
Pathology n (%)	
Papillary	128 (94)
Follicular	6 (4)
Hurthle Cell	2 (2)
PTC histology n (%)	
Classic/conventional	65 (48)
Follicular	35 (26)
Tall cell	18 (13)
Focal tall cell (< 50%)	5 (4)
Oncocytic	9 (7)
Columnar cell	2 (1)
Diffuse sclerosing	1 (0.7)
Hobnail	1 (0.7)
ATA classification n (%)	
High risk	32 (24)
Intermediate risk	78 (57)
Low risk	26 (19)
Post-operative I-131ablation treatment n (%)	
200 mCi	5 (4)
150 mCi	66 (49)
100 mCi	47 (35)
30 mCi	10 (7)
none	8 (6)

### Diagnostic performance of US

A total of 464 US were performed over the course of follow-up, with a mean of 3.4 (SD 2) US per patient. Of the 16/136 patients with cervical recurrence, one did not have US completed at time of recurrence and was excluded from sensitivity and specificity calculations. Based on the historical US reports, there were four (27%) false negative examinations; recurrence was subsequently detected on PET scans and confirmed with pathology in each case. In patients with cervical recurrence, mean time between US and diagnosis of recurrence was 3.5 (95% CI 2.2–4.9 months). Diagnostic accuracy is summarized in Table 2.

**Table 2:** Accuracy of historical ultrasound reports for detecting cervical DTC recurrence

	True recurrence	No recurrence	Total
Positive US finding	11	38	49
Negative US finding	4	82	86
Total	15	120	135

There were 38/136 (28%) patients with at least one US showing false positive abnormalities. Of those, 30 patients with concomitantly stable or undetectable Tg had spontaneous resolution of findings and/or negative biopsy results. The remaining 8 patients had rising Tg, of which, 4 had spontaneous resolution of abnormalities on follow-up (Tg range 0.3–2.6 ng/mL), while 4 subsequently developed distant metastases (Tg range 33.9–445 ng/mL). These false positive US abnormalities resulted in 26 additional specialist follow-up appointments, 13 CT scans, 4 WBS, 4 PET scans, 3 FNAB, and 1 exploratory neck surgery. False positive US were performed in all 13 centres, including 23 US at community centres and 17 US at the academic centre. Overall, the original US interpretations had a sensitivity of 73.3% (95% CI 0.51–0.96) and specificity of 68.3% (95% CI 0.60–0.77) in predicting cervical disease recurrence.

Each true and false positive US were re-interpreted by the subspecialty radiologist. In those without reported findings on US, the most recent US was selected for review to blind the radiologist to results. Re-evaluation using expert review of US utilizing synoptic reporting led to fewer false positive and false negative rates. Diagnostic accuracy is summarized in Table 3. Using these results, US would have a sensitivity of 80% (95% CI 0.60-1.00) and improved specificity of 87.8% (95% CI 0.82–0.93).

**Table 3:** Accuracy of re-interpreted ultrasound for detecting cervical DTC recurrence

	True recurrence	No recurrence	Total
Positive US finding	12	15	27
Negative US finding	3	105	108
Total	15	120	135

### Diagnostic performance of tg

A total of 879 Tg tests were done with a mean of 6.5 (SD 3.2) tests per patient. Diagnostic accuracy is summarized in Table 4. In all patients with true recurrence, serum Tg was detectable (unstimulated Tg median 11.50 [IQR 5.26 − 28.58ng/mL] and stimulated Tg range 10.0–449.3 ng/mL); though one patient had a very low Tg of 0.27 ng/mL at recurrence and we categorized this as “false negative” Tg. Serum Tg was persistently elevated in 5 patients without evidence of structural disease despite a mean of 56 months (SD 13.6) follow-up; these were classified as false positive for specificity calculations. In patients with true recurrence, mean time between Tg measurement and recurrence was 1.06 (95% CI 0.42–1.7 months). The clinical course of these patients is summarized in Table 5. False positive Tg abnormalities resulted in 13 additional specialist follow-up appointments, 7 CT scans, 1 WBS, 3 PET scans, 1 MRI spine, and 2 FNAB. Overall, Tg had a sensitivity of 96.2% (95% CI 0.89-1.0) and specificity of 95.5% (95% CI 0.92–0.99) in predicting any disease recurrence.

**Table 4:** Accuracy of thyroglobulin for detecting local or distant DTC recurrence

	True recurrence	No recurrence	Total
Positive Tg	25	5	30
Negative Tg	1	105	106
Total	26	110	136

**Table 5:**
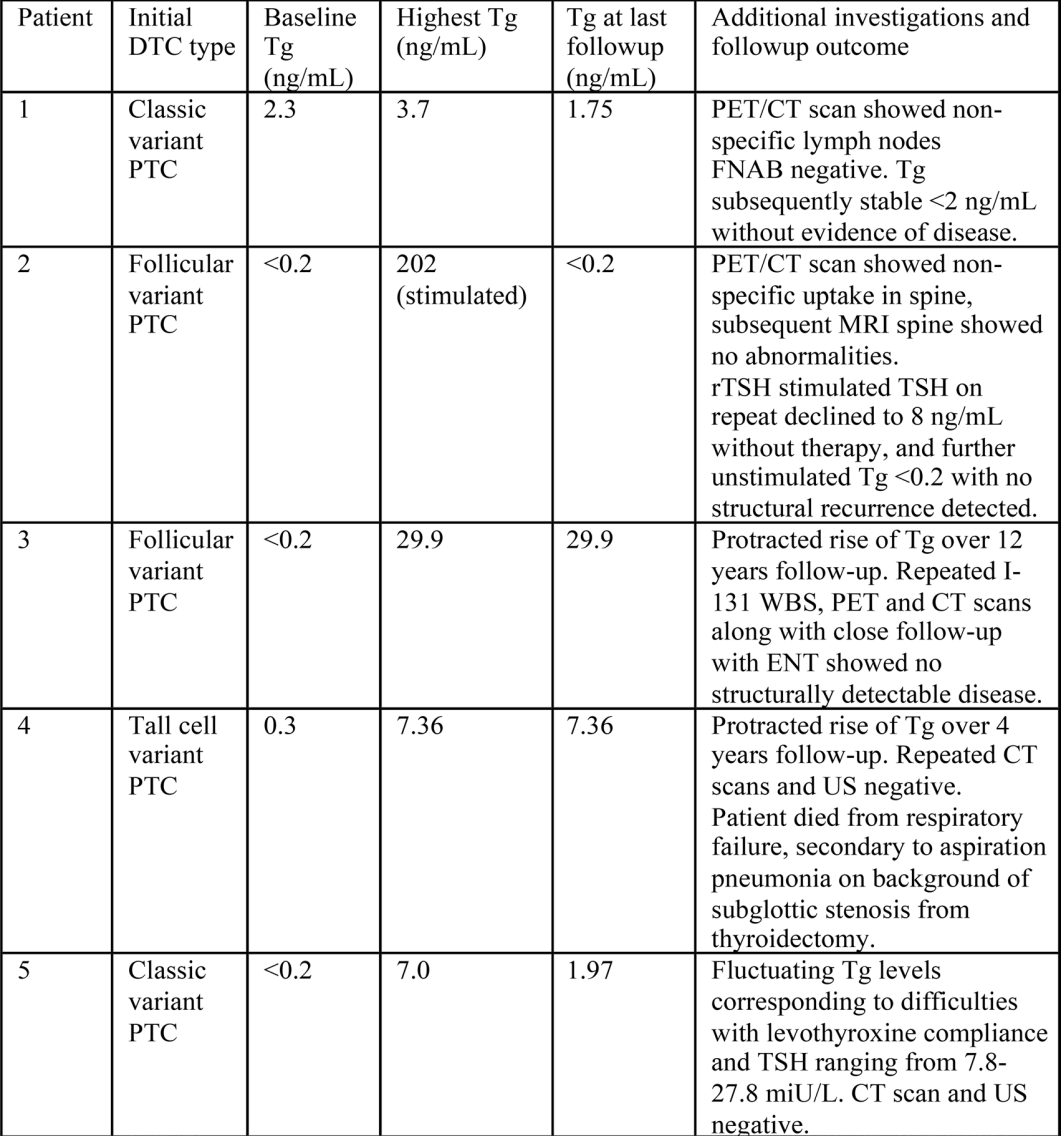
False positive thyroglobulin

## Discussion

While guidelines [3, 4] suggest discontinuing US surveillance in low-risk patients with remnant ablation, negative baseline US, and low serum Tg, in higher risk individuals US is still routinely recommended. This is in part due to the concern of false negative Tg in advanced disease which may be detected on US. However, other studies evaluating ATA low and intermediate risk DTC patients have reported low proportions of patients (1.7–4.8%) with US positive disease but undetectable Tg [8, 10] and it is unclear whether detecting recurrence earlier truly improves survival or quality of life [11]. Our study evaluated a group of higher risk DTC patients, as evidenced by the higher rate of recurrence, and reassuringly demonstrated reliable Tg elevation in 96% of patients with recurrence. Larger studies with a larger population of advanced DTC may help confirm this low risk of false negative Tg, especially with the use of new generation HS-Tg assay.

The optimal frequency and duration of US surveillance remains unclear. For instance, one study [5] suggested that in ATA intermediate risk patients, frequency of US be no more than every 3–5 years in the absence of suspicious clinical features. Another study [12] of lower risk patients found the mean time to recurrence was 19.2 months, with a second increased peak of recurrence at 5–6 years of follow-up; suggesting follow-up US within the first 1–2 years, then a second US at 4–6 years. A large multicentre Korean study [13] also concluded that only 1–2 US within the first 5 years of follow-up is sufficient. They noted that approximately 5% of patients may have had a delay in diagnosis compared to yearly US surveillance but did not correlate this to their clinical status or Tg levels. Our data demonstrated a median time to recurrence of 2.2 years and IQR of 1.4–5.3 years, which may support decreasing frequency of US surveillance particularly after 5 years follow-up, however prospective studies are required to confirm this practice.

False positive US findings continue to be a challenge in long term DTC surveillance. Our study found that having an expert radiologist apply structured evaluation could potentially decrease the number of reported false positive findings; in our study there was a 60% reduction in false positive findings. It is noteworthy that previous studies with even higher false positive rates (34–57%) did have all US performed at specialized academic institutions [5, 6]. Additionally, in a pediatric DTC population with all US performed in a tertiary care academic institution but without synoptic reporting, 55% of patients had at least one falsely indeterminate/suspicious US [14]. In another Canadian study, implementation of ETA guideline-based US reporting resulted in higher quality of reporting, though there was no significant difference in diagnostic accuracy [15]. Within our current healthcare system, which reflects real-world thyroid cancer care seen in most Canadian centres, it would not be realistic to implement centralized US given the limited resources as well as the cost of travel. Given the increased specificity found in our study, requesting a re-interpretation of a positive US examination may be a more feasible alternative. Re-interpretation has shown to alter patient management and be valuable for peer learning in other imaging exams [16].

Even with improvements to US reporting quality, false positive findings continue to exist and lead to earlier specialist follow-up visits as well as additional investigations, which can increase patient anxiety and stress, as well as healthcare costs. DTC patients experience higher rates of depression and anxiety than the general population, and similar health-related quality of life (HrQoL) compared with other cancer patients with worse prognoses [17]. No study has investigated the impacts of such false positive results and ensuing investigations on patients’ HrQoL. The diagnostic accuracy of Tg was excellent in our study; while we conservatively qualified five patients with false positive Tg, two of these have had ongoing rise in Tg and likely have recurrent disease not yet detected on structural imaging. Given the superior sensitivity and specificity of Tg, clinical judgment guided by Tg trend should play a role in the decision making on the appropriate interval of US.

Our study had some limitations. As the AJCC 8th edition staging was introduced in 2018, our study captured stage III and IV patients based on the 7th edition staging, and many would be reclassified with lower staging if examined today. However, the majority (76%) of patients included in our study were intermediate and high-risk patients. Focusing on ATA intermediate and high-risk patients alone in future, larger multi-centred studies would help validate our results. On retrospective review, there was decrease in false positive results by an experienced radiologist using structured evaluation; however, this radiologist was blinded to clinical and biochemical information as well as ability to compare previous imaging which is not reflective of normal practice. While inter-user variability of thyroid nodules has been demonstrated [7], the extent of inter-user variability in post-operative lymph node surveillance is not well described. Our results reflect real-world practices and may not be applicable to centers with specialized and consistent post-operative US reporting. Our findings do not translate to those with hemithyroidectomy or those with anti-Tg antibodies at the time of diagnosis; the frequency and utility of US monitoring in these settings remains unclear. Additionally, the majority of follow-up was done using conventional Tg assay measurements, necessitating use of rTSH-stimulation in early follow-up based on the existing guidelines at the time. Future studies using HS-Tg might further elucidate the true extent of false negative Tg in this population. Finally, the retrospective nature of the study spanned over a long period of time during which some practice patterns have changed.

## Conclusion

In this study of a higher risk cohort of DTC patients, routine US had lower sensitivity and specificity for detecting DTC recurrence compared to serum Tg. Only one patient had recurrence detected by US alone, while the remainder were informed by rising Tg levels. Given the potential psychological burden as well as additional investigations implicated in false positive US findings, we suggest further large-scale studies to elucidate whether routine US is required after the initial 2 years of follow-up in higher risk patients or can be ordered as needed based on Tg levels alone. In our study, standardized evaluation by a subspecialty radiologist improved specificity; request for re-interpretation of indeterminate or positive US findings prior to initiating further investigations may decrease over-investigation and patient distress.

## Data Availability

Data can be made available on reasonable written request to the corresponding author.
